# Dr. Ferguson, on the Deafness of Blacksmiths

**Published:** 1802-05

**Authors:** Andrew Ferguson

**Affiliations:** Aberdeen


### Dr. Ferguson, *on the Deafness of Blacksmiths.*

### To Dr. BRADLEY.

Sir,

THERE has occurred to me of late ſeveral caſes of perſons affected with Dyſecoea, which evidently appeared to be occaſioned by the nature of their employment. Noiſe, eſpecially when violent and ſudden, has been long conſidered, though not the moſt frequent, as one of the cauſes of deafneſs; but I do not recollect of ever hearing before that Blackſmiths were particularly ſubject to any ſpecies of deafneſs the conſequence of their employment; and ſhould perhaps have remained ignorant of this, had not four caſes occurred to me, all of which had a great ſimilarity to each other. The deafneſs had creeped on them gradually. At firſt they became inſenſible to weak impreſſions of ſound, and were not ſo perceptible themſelves of this dulneſs, as thoſe with whom they held converſation. As the deafneſs increaſed, they felt a ringing and noiſe in their ears, which was more or leſs violent at different times; this was ſometimes attended with ſlight vertigo, and pain in the line of the ſutures of the cranium. In one of the caſes the pain was ſomewhat periodical, and very violent. Two of the perſons were between thirty and forty years old, and two of them aged fifty; they were ſtrong men, and excepting this complaint of deafneſs, were otherwiſe healthy. That I might be convinced that the deafneſs was not occaſioned by wax, foulneſs, or any ſubſtance obſtructing the action of the air upon the membrana tympani, I ſyringed their ears with ſoap and water, which I think is moſt proper for this purpoſe; but neither wax, nor any other ſubſtance, could be perceived in the water which was returned from the ear. The patients themſelves obſerved that their ears were in general dry, and that when they picked them, no wax could be perceived. I therefore conſidered the deafneſs to be owing to a paralytic ſtate of the auditory nerves, occaſioned by the noiſe of forging, to which all of them had been in the daily practice of, for many years. One of the caſes was treated as if it had proceeded from this cauſe. Electricity, bliſtering, ſpirituous applications to the cranium, and the injecting into the ears oxygenous air, were the means employed. This being aided by his leaving off the blackſmith buſineſs, was attended with the effect of reſtoring his hearing. The other three perſons were under the neceſſity of continuing at their employment, and therefore little was attempted for their recovery. By giving this a place in the Medical and Phyſical Journal, you will much oblige,

*Aberdeen, Jan.* 1802. ANDREW FERGUSON.

